# A simplified protocol for performing MAGMA/H-MAGMA gene set analysis utilizing high-performance computing environments

**DOI:** 10.1016/j.xpro.2021.101083

**Published:** 2022-01-11

**Authors:** Siwei Zhang

**Affiliations:** 1 Center for Psychiatric Genetics, NorthShore University HealthSystem, Evanston, IL 60201, USA; 2 Department of Psychiatry and Behavioral Neurosciences, University of Chicago, Chicago, IL 60637, USA

**Keywords:** Bioinformatics, Genetics, Genomics, Neuroscience

## Abstract

Here, we present a quick-start protocol to perform generalized gene-set analysis of GWAS data on a metaset of gene lists generated by upstream pipelines, such as differential expression analysis, using the Multi-marker Analysis of GenoMic Annotation (MAGMA) software package and Hi-C coupled H-MAGMA annotation data (de Leeuw et al., 2015; Sey et al., 2020). We specifically tailor the steps and operations to meet the multithreading capability in modern computers, a feature nowadays shared by personal computers and high-performance clusters alike.

For complete details on the use and execution of this profile, please refer to Yao et al. (2021).

## Highlights

• An easy-to-use protocol for entry-level users to perform MAGMA/H-MAGMA analysis
• Scalable; allows analysis on both personal computers and high-performance environments
• Graphing is provided in tutorial to help users in visualizing and interpreting results

## Before you begin

**Timing: hours to days; factors that affect the timing include the number of samples/GWAS cohorts and available computing resources. For each step, intermediate data can be stored for more flexible scheduling on human and computing resources allocation.**

1. The user is expected to have performed the gene expression analysis (or other gene enrichment analyses) and prepared one or more lists of genomic intervals of interest *a priori.* Such lists are usually the results of differential gene expression (DE) analysis packages such as EdgeR (Robinson et al., 2010) and DESeq2 (Anders and Huber, 2010) in R; GO term analysis topGO in R (Alexa et al., 2006), online tools Gene Ontology (GO) knowledgebase (Ashburner et al., 2000; The Gene Ontology Consortium, 2021), DAVID (Huang da et al., 2009); or ChIP-seq/ATAC-seq-specific analysis packages DiffBind (Ross-Innes et al., 2012). When using intervals (peaks), the user should take extra caution to ensure the consistency of the genome version used in the intervals and GWAS association files since most human-based GWAS association data files are still based on the somewhat antiquated GRCh37/hg19 version.
2. This protocol requires a Unix-like environment to run. For computers with Unix-like operation systems installed, most distributions after the Fedora 19 or Debian 7 branches would work. Computers with at least MacOS 10.10 Yosemite installed would work. For computers pre-installed with Windows 10, users can install Ubuntu 20.04 LTS from the in-built Microsoft Store and deploy packages/run commands from within.
3. Steps 1 and 2 will generally require a minimum of 8 GB memory. For Step 3 and later, see the detailed instructions associated with the section itself.
4. Before commencing, it is strongly recommended to use a Python distribution/package manager such as venv/Miniconda/Anaconda to install and manage any dependencies that are used by creating virtual environments.
5. Download MAGMA and H- cAGMA from a credible source, preferably from its distributor site. The distributor sites have been listed in the key resource table.

**Figure 1:**
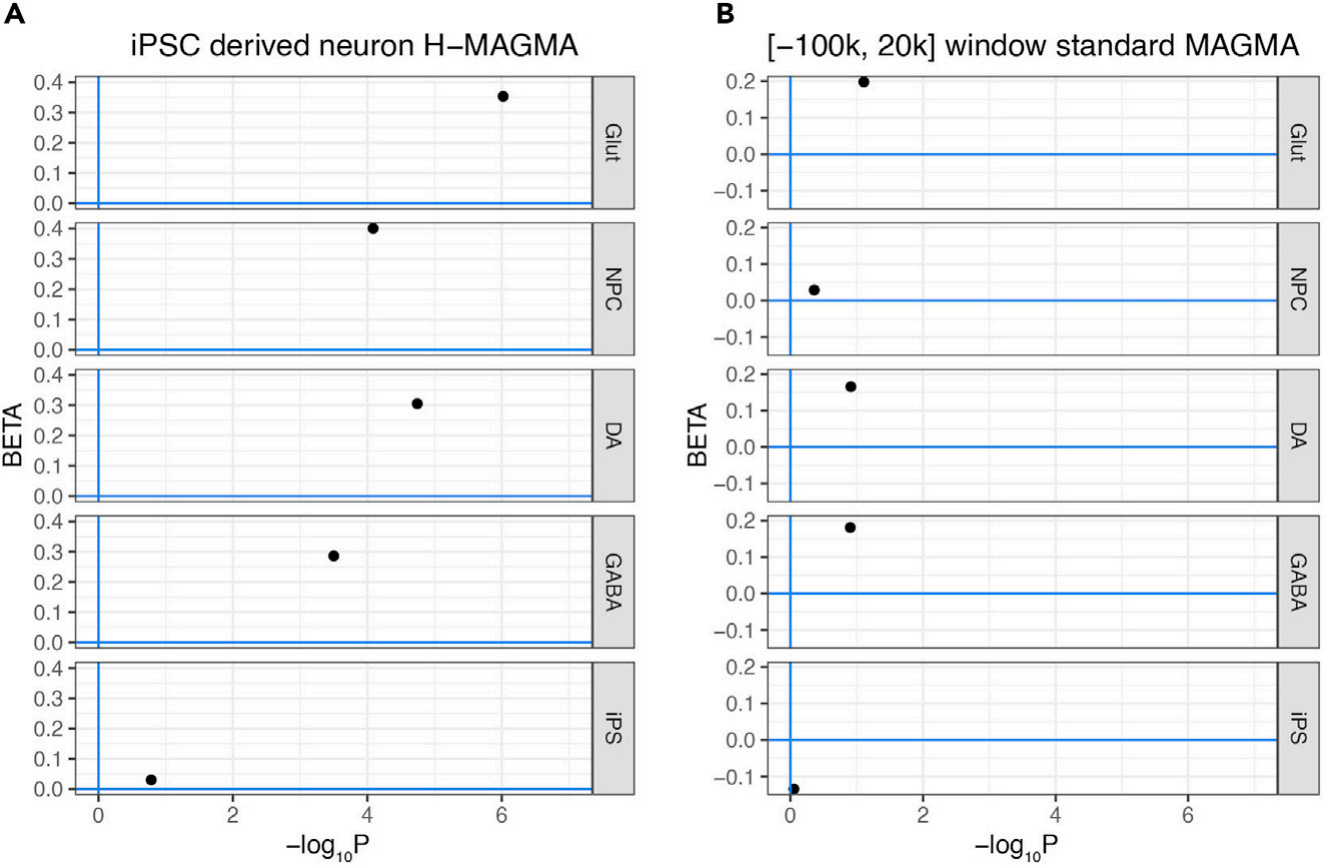
Comparison of H-MAGMA and standard MAGMA analysis results showing the enrichment of schizophrenia risk (PGC wave 3) in *MIR137* target genes in each neuronal cell type but not in the gene set shared with iPS cells XY plots depict each gene set’s corresponding effect sizes (BETA) and enriched p-values (in -log_10_ scale). (A and B) Analysis output of H-MAGMA using iPSC-derived neuron Hi-C gene annotation; (B) Analysis output of standard MAGMA using a slide window size of [-100 kb, 20 kb] and PGC wave 3 gene annotation. Note that the H-MAGMA annotation results have a higher enrichment level than the standard MAGMA analysis using the PGC wave 3 GWAS data, showing its advantage.

***Optional:*** If the user wants to generate graphs as shown in Figure 1, an R environment (R > 4.0) with appropriate libraries (readr > 1.3.1, ggplot2 > 3.3.0, RColorBrewer > 1.1) will be required.

### Create a new virtual environment

**Timing: 30 minto2 h(depending on whether Miniconda has been pre-installed)**

6. Check the presence and install Anaconda/Miniconda on the system.
  a. Open a terminal window or connect to the host through an SSH terminal, type in:
    conda -V
    If there is any returning output similar to “conda 4.10.1”, then it means that an anaconda/Miniconda package manager has been pre-installed with the system. Skip directly to 2. Otherwise, proceed with step b to install the Miniconda package manager.
    ***Note:*** some university-operated computing clusters use their own package managers to load/unload package modules, consult system administrator before processing.
  b. Download and install the Miniconda on the system.
    i. Download the latest Miniconda installer suitable for your system from https://docs.conda.io/en/latest/miniconda.html (Note, for Ubuntu users on Windows 10, please download the Linux 64-bit installer).
    ii. Run the installer by executing the following command after downloaded the installation package in its directory
      bash ./Miniconda3-latest-Linux-x86_64.sh
      and answer the questionnaires for the installer to finish.
    iii. Close and reopen the terminal window for the installer to take effect.

7. Create a new conda environment and install necessary packages.
  a. Create a new conda environment.
    In a new terminal window, type in:
    conda create --name magma_analysis python=3.8
    Here we created a new conda environment with the name magma_analysis and installed Python 3.8 to this environment. To activate this environment, we type:
    conda activate magma_analysis
    You will note that the first part of the command prompt changes to your Miniconda environment directory, such as (/home/your-name/conda-envs/magma-analysis).
  b. Install necessary packages for MAGMA analysis.
    i. Install GNU parallel.
      In the active conda terminal window, type in
      conda install -c conda-forge parallel
      This will install the latest version of GNU parallel and any necessary dependencies associated with it.
    ii. Download and configure MAGMA.
      Go to https://ctg.cncr.nl/software/magma and download the corresponding release version of MAGMA. Note: for Fedora-derived systems, you have to compile the binary file from the source.
      Unzip the download file and place the main binary file (magma) in a directory of your preference (e.g., /software/magma/). If preferred, you may add the path of this directory as part of the $PATH in your .bash_profile for convenience. Remember to reopen the terminal window or source the .bash_profile for the change to take effect. When all configurations have been set, open a console terminal and execute the following codes to verify that magma has been properly installed (assuming you have configured the magma path in $PATH):
      conda activate magma_analysis
      magma --version
      Then the console should return something similar to:
      MAGMA version: v1.08 (custom)
    iii. Download the MAGMA auxiliary files.

It is also handy to download the gene location data, SNP synonyms, and reference data files from Phase 3 of the 1000 Genomes Project (Genomes Project Consortium et al., 2015) for subsequent use (included in the Deposited data section of the key resources table). Note: all the data listed on this page were built based on GRCh37/hg19 genome. Hence, if you have any coordination data based on the more recent GRCh38/hg38 genome, you have to either perform a genome liftover or find the corresponding hg38 version of the reference files (the GitHub repository of this article includes an introduction on downloading and creating a set based on the 1000G European population). Place the auxiliary files in a separate path from the MAGMA executable (e.g., /data/your-name/magma_aux_files/).

## Key resources table

**Table:** 

REAGENT or RESOURCE	SOURCE	IDENTIFIER
**Deposited data**		

Original data used to create Figure 4C in Yao et al. (2021)	Yao et al. (2021)	https://ars.els-cdn.com/content/image/1-s2.0-S2589004221007537-mmc2.xlsx
PGC Schizophrenia wave 3 GWAS 2021	The Schizophrenia Working Group of the Psychiatric Genomics Consortium et al. (2020)	https://doi.org/10.6084/m9.figshare.14672178
Instruction on obtaining and making the GRCh37/hg19 or GRCh38/hg38 version of 1000G EUR plink file set (SNP_Loc_File)	This article	https://github.com/endeneon/MAGMA_analysis_protocol/tree/main/g1k_eur_hg38
Revised gene location file (in GRCh37/hg19) containing gene symbols as IDs (Gene_Loc_File),	This article	https://github.com/endeneon/MAGMA_analysis_protocol/blob/main/data_files/Rev.NCBI37.3.gene.loc

**Software and algorithms**		

MAGMA software, gene location, and GRCh37/hg19 plink population file sets		https://ctg.cncr.nl/software/magma
H-MAGMA codes and annotation files		https://github.com/thewonlab/H-MAGMA
Miniconda		https://docs.conda.io/en/latest/miniconda.html

## Step-by-step method details

We will recapitulate the H-MAGMA analysis in Figure 4C of Yao et al. (2021), with an added section of standard MAGMA analysis using a user-generated annotation file instead of the gene annotation file provided by H-MAGMA from Hi-C data.

### Run gene annotation

**Timing: 30 minto1 h. Store intermediate data for more flexible scheduling on human and computing resources allocation.**

Here we will start with a clean slate by directly using the .bim file (the SNP location file of the plink bim/bed/fam file sets) associated to the g1000_eur data set (SNP_Loc_File, download links included in KRT) and one revised version of the gene location file from the original MAGMA gene location build 37 file (Gene_Loc_File, download links included in KRT). As an example, we could use the .bim file (such as the g1000_eas.bim or g1000_eur.bim files from the 1000 Genome Project, downloadable in KRT) as the SNP_Loc_File and the Rev.NCBI37.3.gene.loc (also in KRT) as the Gene_Loc_File as a start.

1. Pre-run preparations.
  a. Confirmation of data format compatibility. It is of utmost importance that all files use the same naming rules during the analysis since different data files, especially reference data files, may be acquired from distinct sources. The common fields that could cause incompatibility and therefore error during analysis include, but are not limited to:
    i. Chromosome names: check the first few lines of both SNP_Loc_File and Gene_Loc_File and confirm whether the chromosome names are consistent (e.g., both “1” or “chr1”). Since the SNP_Loc_File (.bim) file usually comes with chromosome names in 1, 2, …, in most cases it would be the Gene_Loc_File that has an excessive “chr” prefix to the chromosome names. To remove the prefix, simply run the following bash script:
      awk -F '\t' '{gsub("chr","",$2)}1' Gene_Loc_File > New_Gene_Loc_File
    ii. Gene names: Different Gene_Loc_File may use different strings (gene symbols, ENSEMBL gene ID, NCBI gene ID, etc.) in the first column of Gene_Loc_File. This column needs to be consistent with the names used in the gene set file (Set_File) that will be mentioned later. If different gene symbols (such as ENSEMBL gene symbols vs UCSC gene names) were used between the two files, some lookup table-based conversion may need to be performed. Some basic (but very handy) conversion can be performed on g:Profiler (https://biit.cs.ut.ee/gprofiler/convert) and the user could consult additional web resources, such as R biomaRt package, for more in-depth study themselves.
    iii. (Rare) 0-based or 1-based gene tracks. In some rare cases when the gene annotation file (Annot_File) was pre-generated rather than made de novo (as in the case of H-MAGMA), a 0-based Annot_File might be used together with a 1-based Set_File (especially when genomic intervals were used instead of gene names). In order to avoid such misadventure, it is recommended to spot-check a few known SNP and genes for their positions to see if they are consistent.
  b. Decide the size of the annotation window around genes.

In order to include up/down-stream SNPs into genes of interest for analysis, it is necessary to set a sliding window around the genes using the --annotate flag. Although there is no universally agreed value in setting the size of the sliding window, users are recommended to check related published results for optimized results.

(!) Warning: if the ignore-strand modifier is used, all genes will be assumed to be located on the positive (+) strand with the strand column information ignored. Use with caution.

2. Run the annotation.

It is recommended to write and save each command as a separate bash script for easy debugging and code recycling using editors such as Vim, GNU Emacs, or VS Code.

It is also recommended to pre-define the location of all data files as variables instead of embedding them in the magma command line, for easy code recycling.

Assuming that the magma executable path has been added to your $PATH variable.

Here we will run a standard annotation using window size of 100 kb upstream, 20 kb downstream, output file prefix is miR137_100k_20k and the output file will be sent to the current directory. If performing H-MAGMA analysis rather than the standard MAGMA model, skip from here to step 3. Save the following code as gene_annotation.sh.

#!/bin/bash

SNP_Loc_File="/data/your-name/magma-aux-files/SNP_Loc_File"

Gene_Loc_File="/data/your-name/magma-aux-files/Gene_Loc_File"

Output_Prefix="miR137_100k_20k"

magma \

 --annotate window=100,20 \

 --snp-loc $SNP_Loc_File \

 --gene-loc $Gene_Loc_File \

 --out $Output_Prefix

***Note:*** users should carefully choose the path of “/data/your-name/magma-aux-files/” section since they need to define this part themselves. The Output_Prefix is customizable as long as it is kept consistent in all subsequent steps. This rule applies in all subsequent steps and associated code blocks.

Then execute the following command in the console.

bash ./gene_annotation.sh

After this operation, the user will find two files: miR137_100k_20k.genes.annot and miR137_100k_20k.log generated under the current directory. The .log file recorded the actual commands that were executed as well as some details during the program execution. Nevertheless, here we focused on the generated.genes.annot file (Annot_File) for the next step of gene analysis. If we examine the content of the Annot_File, we will find a row-based file that each gene was marked by an interval of its length plus the window size, followed by the id of all the SNPs that fall within the interval.

### Gene analysis

**Timing: 30 minto1 h**

During this step, we will calculate the p-values and associate metrics of each gene and store them in the generated .genes.out and .genes.raw files for subsequent gene-level analysis uses. Moreover, we will take advantage of the parallel computing ability of modern computers/clusters to boost the process to a significantly faster degree.

3. Identify and confirm the available system resources to use.
  a. Identify the numbers of system CPUs/threads.
    In the console, type in:
    lscpu | grep "CPU"
    Then the system should return a few lines of system information. The content may vary depending on the computer/cluster model and configuration. Nevertheless, the general format should look like this:
    What we are looking at here is the second line. Here we have 64 logical CPUs/threads at our disposal for the subsequent parallel run.
    CPU op-mode(s):    32-bit, 64-bit
    CPU(s):         64
    On-line CPU(s) list:  0-63
    CPU family:       6
    Model name:      Intel(R) Xeon(R) Gold 6226R CPU @ 2.90GHz
    CPU MHz:         3019.667
    CPU max MHz:       3900.0000
    CPU min MHz:       1200.0000
    NUMA node0 CPU(s):     0-15,32-47
    NUMA node1 CPU(s):     16-31,48-63
  b. Identify the size of available memory to use.

In the console, type in:

free -h

The console returns:

     total   used   free shared buff/cache available

Mem: 503Gi 40Gi  5.7Gi  920Mi     456Gi   458Gi

Swap: 35Gi  3.8Gi 32Gi

So we can see there are 458 GiB of memory at our disposal.

4. Compose the main code part for gene analysis using SNP p-value data with parallel processing.
  a. Verify GNU Parallel is installed on the system.
    In the console, type in:
    parallel --version
    See if the console successfully returns the current version of the parallel installed.
  b. Calculate the appropriate number of parallel threads to use.
    As a general rule, each MAGMA session would use approximately 1 GiB of memory. Hence, the maximum theoretical parallel thread numbers we can set will be the size of available memory divided by 1 GiB. However, we should also consider the fact that the number of parallel threads cannot exceed the count of logical cores of the system, i.e., the number we collected from step 3a. Also, we need to reserve some resources for system use. Hence, it is generally recommended that the number of parallel threads to use should not exceed 10 on computers < 16 logical cores and not exceed 40 in mainframe clusters, as long as the user-available memory permits.
  c. Prepare a temp working folder for storing intermediate batch files and write the code. Here we will use the data file from the Psychiatric Genomics Consortium Schizophrenia Wave 3 (scz2021) (The Schizophrenia Working Group of the Psychiatric Genomics Consortium et al., 2020) as the SNP_Pval_File). The download link has been included in the key resource table.

In a text editor, type in the following code and save it as gene_analysis.sh

#!/bin/bash

mkdir temp_annot # make a temporary directory to host the intermediate files

Data_File="/data/your-name/magma/aux-files/g1000_eur"

Annot_File="miR137_100k_20k.genes.annot"

SNP_Pval_File="/data/your-name/PGC3/PGC3_SCZ_wave3_public.2021.v2.tsv"

Output_Prefix="miR137_PGC_SCZ_w3"

# run magma in parallel, 8 threads in this case

parallel magma \

 --batch {} 8 \

 --bfile $Data_File \

 --gene-annot $Annot_File \

 --gene-model snp-wise=mean \

 --pval $SNP_Pval_File ncol=Nca \

 --out temp_annot/$Output_Prefix \

 ::: {1..8}

# merge all intermediate files generated under the temp_annot files

# and send out for one single file set

magma \

 --merge temp_annot/$Output_Prefix \

 --out temp_annot/$Output_Prefix

# extract merged files for subsequent analysis

cp temp_annot/$Output_Prefix.genes.∗ .

# remove the temporary directory

rm -r temp_annot

Then execute the following command in the console.

bash ./gene_analysis.sh

This snippet will automatically perform a series of operations as the in-line comments have noted. Finally, it will generate three files started with the Out_Prefix: a .genes.raw file contains the raw permutation results, a .genes.out file contains the per-gene statistics, and a .log file records the commands used.

Users may want to tweak the gene analysis model used during analysis and see if which one fits their data better. Indeed, there are three base analysis models in MAGMA: the principal components regression (linreg) model, the SNP-wise mean (snp-wise-mean) model, and the SNP-wise Top 1 (snp-wise=top) model.

Here we choose the SNP-wise mean model for our PGC Schizophrenia analysis since we know that mental disorders tend to be of cumulative, non-mendelian inheritance, therefore a model that is more attuned to the mean SNP association and skews towards associations in areas of higher LD in individual genes is preferable. However, the in-depth discussion on the statistical basis and their associated disease application would be too complex and beyond the scope of this protocol. For further discussion on all the modifiers, their statistical basis, and the potential impact on the performance, we recommend the user read the official MAGMA manual and original publications.

Users should also note the N/ncol modifier under the --pval option that is used to specify the sample size. First, not all the SNP_Pval_File has the sample size column. In these cases, the sample size is usually stated in the associated README file and users should specify N = [sample_size] (e.g., N = 25350) instead of using the ncol modifier. Secondly, SNP_Pval_File from different sources may have different names for their sample size column (e.g., Nca, Neff, Ngt). User should manually check the first few lines of the SNP_Pval_File file and confirm the column name before applying in code.

The MAGMA user manual contains a detailed explanation of the structures and columns of the generated .genes.out file. Users may want to refer to the corresponding section for the information contained in each column if interested.

**CRITICAL:** If performing H-MAGMA analysis instead of standard MAGMA analysis, it is crucial to use the H-MAGMA provided Annot_File corresponding to your cell/tissue of interest instead of the one generated in step 2.

### Gene set analysis

**Timing: 15–30 min**

In this section, we will integrate the gene-level analysis results derived from the previous section together with a list containing one or more gene sets for a linear regression model-based test.

***Note:*** the original H-MAGMA package provided input files using gene names in ENSEMBL gene ID without the sub-version numbers, which are different from the record in most GENCODE or ENSEMBL GTF annotation files (e.g., ENSG00000153266 instead of ENSG00000153266.13). Users are advised to verify their own gene list to make sure they are compatible.

5. Prepare the gene set list file.

The gene set (Set_Annot_File) could be written in either row-based or column-based format (see Appendix A of the MAGMA manual for details). From our experience, the column-based format allows greater versatility and better clarity when working in conjunction with other bioinformatic package outputs, although using the column-based input file format does require the addition of the “col” modifier (see below). Another note is each line of the Set_Annot_File needs to terminate with the newline (\n) character only but not the Windows carriage return (\r\n), hence, some tab-delimited files exported directly from Microsoft Excel in Windows may not work before having their \r character stripped.

6. In a text editor, type in the following code and save it as gene_set_analysis.sh

#!/bin/bash

Gene_Results_File="miR137_PGC_SCZ_w3.genes.raw"

Set_Annot_File="/data/your-name/analysis-results/miR137_gene_set_list_4_magma.txt"

Output_Prefix="miR137_100k_20k_gene_set_results"

magma \

 --gene-results $Gene_Results_File \

 --set-annot $Set_Annot_File col=2,1 \

 --out $Output_Prefix

Then execute the following command in the console.

bash ./gene_set_analysis.sh

**Figure 2:**
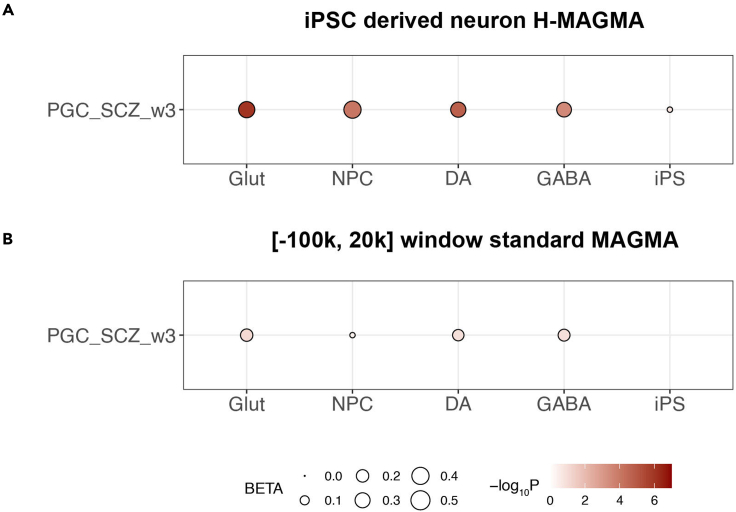
Data from Figure 1 shown in bubble plot format (A and B) Analysis output of H-MAGMA using iPSC-derived neuron Hi-C gene annotation; the diameter of points corresponds to each BETA value and the shade of points corresponds to each -log10P value of the sample shown. Note that the iPS have a negative beta value hence no point was plotted in the panel.

We also provided another file set for the readers to recapitulate the analysis in the Figure 4C of Yao et al. (2021) using the iPS-derived neuron Hi-C data from H-MAGMA (Figure 2A). The corresponding file set can be found in the GitHub repository of this article.

The structure and columns of the output .gsa.out file has been well-documented in the MAGMA manual. Essentially, we will need the beta (regression coefficient) and P values to see if our different gene sets are more concordant to our disease model (PGC3 schizophrenia here).

***Optional:*** Make bubble plots using R and ggplot2 to show beta and enrichment value.

**Timing: 15–30 min**

#!/usr/bin/Rscript

# init

library(readr)

library(ggplot2)

library(RColorBrewer)

# plot panel B

df_raw <- read_table2("Apr122021_set_analysis.gsa.out",

             skip = 4)

df_raw$`-log10P` <- 0 - log10(df_raw$P)

df_raw$VARIABLE <- factor(df_raw$VARIABLE,

levels = c("Glut", "NPC", "DA", "GABA", "iPS"))

df_raw$TYPE <- "PGC_SCZ_w3"

ggplot(df_raw, aes(x = VARIABLE,

            y = TYPE,

            size = BETA,

            fill = `-log10P`)) +

scale_fill_gradient(low = "white",

            limits = c(0, 7),

            high = "darkred") +

scale_size_area(limits = c(0, 0.5)) +

geom_point(shape = 21) +

xlab("Cell type") +

ylab("") +

theme_bw() +

theme(legend.position = "bottom",

    legend.box = "horizontal",

    axis.text = element_text(size = 14),

    axis.title = element_text(size = 16))

To replot the Figure 4C of Yao et al. (2021), create a new R project and a new R code window in RStudio. Ensure that library ggplot2and RColorBrewer has been installed. Then type in:

Then execute the whole code block in RStudio (select-all then ctrl + ENTER) or execute line-by-line (ctrl + ENTER). The generated ggplot2 graph will appear in the plot window of the RStudio. This will generate panel B in Figure 2. To generate Figures 1 and 2A, use the code provided in the GitHub repository.

## Expected outcomes

Here we can compare the two sets of outputs, one standard MAGMA results using sliding window size of 100 kb, 20 kb size, the other using H-MAGMA model from iPS-derived neuron Hi-C data (Rajarajan et al., 2018) from the same five sets of genes in the Table S1 of Yao et al. (2021) (Figures 1 and 2, an organized, ready-to-use version was also provided in the associated GitHub repository). The raw .gsa.out files were provided on GitHub.

## Limitations

As noted above, there has not been a universally accepted value of the sliding window size around genes and this may need several rounds of optimization. In addition, there are some recent discussions related to the statistical stability of the H-MAGMA analysis and possible fallouts (de Leeuw et al., 2020; Yurko et al., 2021). Users are advised to explore these discussions and make the best decisions for their specific cases.

## Troubleshooting

### Problem 1

MAGMA pipeline fails at merge (step 4c)

In some rare cases, MAGMA returns a merging error after finishing the parallel gene analysis step. It is usually caused by one of the many parallel threads that unexpectedly failed during execution, thus resulted in a missing part in the generated file series.

### Potential solution

In most cases, simply re-run the bash snippet should solve the issue. If the issue still remains unsolved, comment out the rm -r temp_annot line at the end of the snippet and investigate the content of temp_annot. Check if there is any missing entry in the consecutive numbering of $Output_Prefix_(method).batch(X)_N.genes.out files. If the missing number was found as X, step back to the parent directory and execute:

magma \

 --batch X N \

 --bfile $Data_File \

 --gene-annot $Annot_file \

 --gene-model snp-wise=mean \

 --pval $SNP_Pval_File ncol=Nca \

 --out temp_annot/$Output_Prefix

Here N will be the same number of threads (e.g., 8) used in the previous snippet and this one-line will generate the single missing file. Then proceed with merging:

### Problem 2

In some cases, the GWAS reference file may contain malformed lines. These lines may cause errors in some versions of MAGMA (malformed line numbers will be reported in the MAGMA output).

### Potential solution

Open the text file in a text editor (e.g., VIM) and manually remove malformed lines. The line number option in VIM can be turned on by using :set number command

magma \

 --merge temp_annot/$Output_Prefix \

 --out temp_annot/$Out_Prefix

## Resource availability

### Lead contact

Further information and requests for resources and reagents should be directed to and will be fulfilled by the corresponding author (szhang@northshore.org).

### Materials availability

No biological reagents are required as part of this protocol.

## Data Availability

The dataset used in Yao et al. (2021) is available for download as supplementary files of the original research paper. For the GWAS database used and additional sources, please refer to the key resource table. Additional data files, scripts, and sample output can be found at the GitHub repository for this protocol at https://github.com/endeneon/MAGMA_analysis_protocol.
